# The Desmoplastic Stroma Plays an Essential Role in the Accumulation and Modulation of Infiltrated Immune Cells in Pancreatic Adenocarcinoma

**DOI:** 10.1155/2011/212810

**Published:** 2011-12-06

**Authors:** Vegard Tjomsland, Lina Niklasson, Per Sandström, Kurt Borch, Henrik Druid, Charlotte Bratthäll, Davorka Messmer, Marie Larsson, Anna Spångeus

**Affiliations:** ^1^Division of Molecular Virology, Department of Clinical and Experimental Medicine, Linköping University, 581 85 Linköping, Sweden; ^2^Division of Surgery, Department of Clinical and Experimental Medicine, Linköping University, 581 85 Linköping, Sweden; ^3^Department of Oncology-Pathology, Karolinska Institutet, 171 77 Stockholm, Sweden; ^4^Division of Oncology, Kalmar Hospital, 391 85 Kalmar, Sweden; ^5^Moores Cancer Center, University of California San Diego, La Jolla CA 92093, USA; ^6^Division of Internal Medicine and Department of Endocrinology, Department of Medical and Health Science, Linköping University, 581 85 Linköping, Sweden

## Abstract

Tumor microenvironment is composed of tumor cells, fibroblasts, and infiltrating immune cells, which all work together and create an inflammatory environment favoring tumor progression. The present study aimed to investigate the role of the desmoplastic stroma in pancreatic ductal adenocarcinoma (PDAC) regarding expression of inflammatory factors and infiltration of immune cells and their impact on the clinical outcome. The PDAC tissues examined expressed significantly increased levels of immunomodulatory and chemotactic factors (IL-6, TGF**β**, IDO, COX-2, CCL2, and CCL20) and immune cell-specific markers corresponding to macrophages, myeloid, and plasmacytoid dendritic cells (DCs) as compared to controls. Furthermore, short-time survivors had the lowest levels of DC markers. Immunostainings indicated that the different immune cells and inflammatory factors are mainly localized to the desmoplastic stroma. Therapies modulating the inflammatory tumor microenvironment to promote the attraction of DCs and differentiation of monocytes into functional DCs might improve the survival of PDAC patients.

## 1. Introduction

Many types of tumors have an inflammatory microenvironment comparable to what is found in chronic inflammatory responses, that is, are enriched in inflammatory cells and mediators, transformed tissue, and increased angiogenesis [1]. The inflammation is created by the interplay between tumor cells and the surrounding stroma, for example, cancer associated fibroblasts (CAF), immune cells, and extracellular matrix and gives rise to an environment favoring tumor expansion [2]. The clinical relevance of the microenvironment regarding tumor progression is supported by the correlations seen between poor outcome and CAFs, angiogenesis and the composition and amount of infiltrating inflammatory cells [3].

 Pancreatic ductal adenocarcinoma (PDAC) is a common gastrointestinal malignancy characterized by rapid progression, resulting in poor outcome and a 5-year survival rate of less than 5% [4]. Like in most adenocarcinomas, PDAC has a massive fibrotic stoma, that is, desmoplasia [5–7], which contributes to the local inflammatory environment at the tumor site as well as systemically [8]. The microenvironment found in PDAC supports tumor growth, progression, and the recruitment of leukocytes, such as macrophages, dendritic cells (DCs), T cells, and neutrophils [9–11]. Infiltration of these cells has been detected in a variety of cancers [12, 13]. Several studies have reported that blood DCs and tumor infiltrating DCs exhibit phenotypic and functional abnormalities when isolated from tumor bearing animals and patients with PDAC [14–16]. Given their pivotal role in the adaptive immunity and tumor surveillance in healthy individuals, this impairment might contribute to the tumor escape from the immune system [17]. Increased numbers of DCs have been associated with improved outcome in various types of human cancers, and some studies have also pointed out the DC maturation as a prognostic indicator [18]. We have previously observed a correlation between survival time for PDAC patients and the amount and phenotype of blood DCs, which implicate the importance to maintain a functional DC compartment [16, 19]. Different inflammatory mediators, for example, cyclooxygenase-2 (COX-2), IL-1, IL-6, TGF*β* and CXCL8, and their receptors are present in the tumor milieu [20–22]. COX-2 is expressed by several solid tumors, including PDAC, and correlates with tumor invasion and clinical outcome [22]. Moreover, COX-2 is believed to have an influence on the DC impairment, and recent findings have provided evidence that the COX-2 metabolite prostaglandin E_2_ (PGE_2_) is involved in the upregulation of indoleamine 2,3-dioxygenase (IDO) in DCs [23, 24]. IDO expression transforms DCs into tolerogenic cells that activate regulatory T cells (Tregs), which have been shown to exist in several types of cancers [13]. 

In the present study we found elevated levels of several inflammatory factors, including CCL2, CCL20, TGF*β*, IDO, IL-6, and COX-2, in the PDAC tissue. Furthermore, PDAC tissues had significantly elevated levels of infiltrating macrophages, cytotoxic T cells, and DCs. Low levels of MDC, PDC, and mature DC markers were associated with poor prognosis. Treatments that direct the inflammatory tumor microenvironment to attract high levels of DCs could be beneficial for the clinical outcome of the PDAC patients.

## 2. Material and Method

### 2.1. Patients and Controls Enrolled in the Study

Tumor tissues were obtained from 30 PDAC patients undergoing Whipple resection at Linköping University Hospital, Sweden. The patients did not receive any neoadjuvant chemo/radiotherapy and the diagnosis was histologically confirmed by two pathologists. The control group consisted of pancreatic tissue from ten individuals, seven individuals deceased from hypothermia and three patients with benign disease (adjacent pancreatic tissue with normal histology was used in the study). The pancreatic tissues were frozen immediately and cryopreserved in liquid nitrogen. Paraffin-embedded tissue sections from patients and controls were obtained from the department of Pathology at Linköping University Hospital, Sweden and the department of Oncology-pathology, Karolinska Institutet, Stockholm, Sweden. The PDACs were staged according to the 1997 International Union against Cancer classification (TNM = Tumor, Node, Metastasis), and the PDAC patients ranged from T1–T4 (T1 (*n* = 3), T2 (*n* = 14), T3 (*n* = 12), and T4 (*n* = 1)), N0 (*n* = 6), N1 (*n* = 24), and M0 (*n* = 30) stage. The study protocol and patient consent documents were approved by the Regional Ethics committee in Linköping, Sweden (Dnr. M38-06).

### 2.2. RNA Extraction and Quantification with Real-Time PCR

Total RNA was prepared from the samples using Trizol (Invitrogen) according to manufacturer's protocol and cDNA synthesized with SuperScript III Reverse Transcriptase First-Strand cDNA Synthesis kit according to the manufacturer's protocol (Invitrogen). Quantitative PCR was performed with Fast SYBR Green Master Mix (Version 09/2007; Applied Biosystems, Foster City, Calif, USA) on 7900 Fast Real-Time PCR system with 7900 System SDS 2.3 Software (Applied Biosystems) according to the manufacturer's protocol. Specific primers for CCL2, CCL20, IL-6, TGF*β*, CD1a, CD1c, CD68, CD163, CD208, CD209, CD303 (CyberGene AB), and COX-2 (Invitrogen) were used. *β*-actin, and Glyceraldehyde-3-phosphate dehydrogenase (GAPDH) (CyberGene AB) were utilized as housekeeping genes. The primers were designed using Primer Express (Applied Biosystems). Real-time PCRs for the detection of IDO and PD1 were performed using TaqMan Gene Expression Assays (Applied Biosystems) according to the manufacturer's protocol. All reactions were performed in triplicates including nontemplate controls and two endogenous control probes. FAM conjugated, gene-specific assays were Hs00984148_m1 (IDO), Hs00169472_m1 (PD1) and the endogenous controls Hs01003267_m1 (HPRT1), and Hs99999905_m1 (GAPDH). All reactions were performed in triplicates including nontemplate controls. The results were analyzed using the ΔΔCt method [25] and the data were presented as the quantitative expression of each gene.

### 2.3. Immunohistochemical Staining of PDAC and Normal Pancreatic Tissues

Formalin fixed, paraffin-embedded samples of tumor tissue from PDAC patients (*n* = 30) and normal pancreatic tissue (*n* = 10) were cut in 5 *μ*m sections. The sections were then rehydrated and antigen retrieval was performed in a microwave oven for 15 min (350 W) using citrate buffer (pH 6.0). Endogenous peroxidase was minimized by 10 minutes incubation in H_2_O_2_, and nonspecific binding was avoided by incubating with Background Sniper (Biocare Medical) or 1% bovine serum albumin for 10 min. The samples were immunostained overnight with antibodies (Ab) for CXCL8 (1 : 25, Becton Dickinson), COX-2 (1 : 200, CRM306B, Biocare Medical), S100 (dilution 1 : 1000, Z 0311, Dako, Sweden), CD163 (dilution 1 : 10, ab74604), CD83 (dilution 1 : 50, ab64875), CD8 (dilution 1 : 600, ab4055), IL-1*α* (1 : 40, ab7632), CCL2 (1 : 1000, ab73680), and CCL20 (1 : 40, ab9829) (Abcam, UK). The sections were then incubated with alkaline phosphatase conjugated anti-mouse or anti-rabbit secondary Ab (Jackson ImmunoResearch) for one hour or by using LSAB2 System-HRP kit (K0675, Dako) containing biotinylated link and streptavidin-conjugated HRP according to the manufacturer's protocol. Alkaline phosphatase was detected by Vulcan fast red chromogen 2 solution (Biocare Medical) according to the manufacture's protocol. HRP was detected by development in Tris-buffer containing diaminobenzidine tetrahydrochloride (DAB) (Saveen-Werner AB) and 10 *μ*L of 30% H_2_O_2_. Counter-staining was performed with methyl green solution (0.1 M sodium Acetate buffer, pH 4.2) containing 1% methyl-green (Sigma Aldrich) or hematoxylin.

### 2.4. Quantification of Inflammatory Factors and Immune Cells in Tumor Tissue

The amount of CD163, CD8, CD83, and S100 immunoreactive cells and the COX-2 ratio between tumor cells and stroma were analyzed using Quantimet 500MC image processing analysis systems linked to a Leica DM LB microscope (Leica Microsystems) supported by Leica QWin software version 3 (Leica Microsystems). CD163, S100, CD83, and CD8 positive cells were manually marked in 20 randomly chosen fields (×40 magnification) using automated standard operation sequences created by QUIPS (Leica Microsystems), an interactive programming system included in the Leica QWin software. The number of immunoreactive cells/*μ*m^2^ pancreatic tissue was calculated. To evaluate the COX-2 ratio between tumor cells and stroma, PDAC tissues were immunohistochemically stained with a combination of rabbit anti-human COX-2 and mouse anti human Ki-67 Ab, followed by alkaline phosphatase- and HRP-conjugated secondary Ab and detected as previously described. Ten randomly selected fields were chosen and areas of double positive cell structures (tumor structures) were marked using an automated standard operation sequence created by QUIPS and compared to nonproliferative COX-2 positive stroma.

### 2.5. Statistics

The statistical analysis was performed with GraphPad Prism 5 (GraphPad Software, La Jolla, Calif, USA). A *P*-value < 0.05 was considered statistically significant and error bars throughout indicate standard error of the mean (SEM). Nonparametric data was analyzed using the Wilcoxon matched pairs test followed by Mann-Whitney test. Survival curve was analyzed by the Kaplan-Meier survival method, and statistical significance was determined using Log-rank (Mantel-Cox) test and *P* value < 0.05 was considered statistically significant.

## 3. Result

### 3.1. PDAC Microenvironment Contains Elevated Gene and Protein Expression Levels of Inflammatory Factors

**Figure 1:**
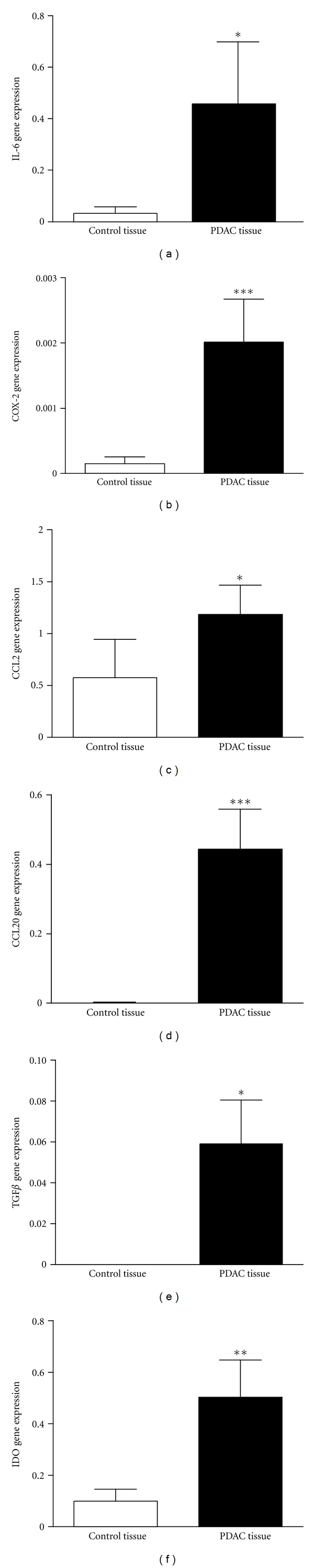
Elevated gene expression levels of inflammatory factors in PDAC tissue. RNA was extracted from PDAC (*n* = 30) and normal pancreatic tissue samples (*n* = 10) and assessed for relative gene expression levels of the inflammatory factors, IL-6 (a), COX-2 (b), CCL2 (c), CCL20 (d) TGF*β* (e), and IDO (f). *P < 0.05, **P < 0.05, ***P < 0.001.

We examined the gene expression levels of several inflammatory factors by qPCR in PDAC tissues and compared them to the levels found in pancreatic tissues from the controls. IL-6 (*P* = 0.023), COX-2 (*P* < 0.001), CCL2 (*P* = 0.035), CCL20 (*P* < 0.001), TGF*β* (*P* = 0.016), and IDO (*P* = 0.003) gene expression levels were all significantly increased in PDAC tissues compared to controls (Figures 1(a)–1(f)). Gene expression of programmed death-1 receptor (PD1) was not detected in any of the control samples (*n* = 10), but detected in 40% of the tumor samples (*n* = 30) (data not shown).

### 3.2. Distribution of Inflammatory Factors in Tumor Cells and Stroma in PDAC Tissues

**Figure 2:**
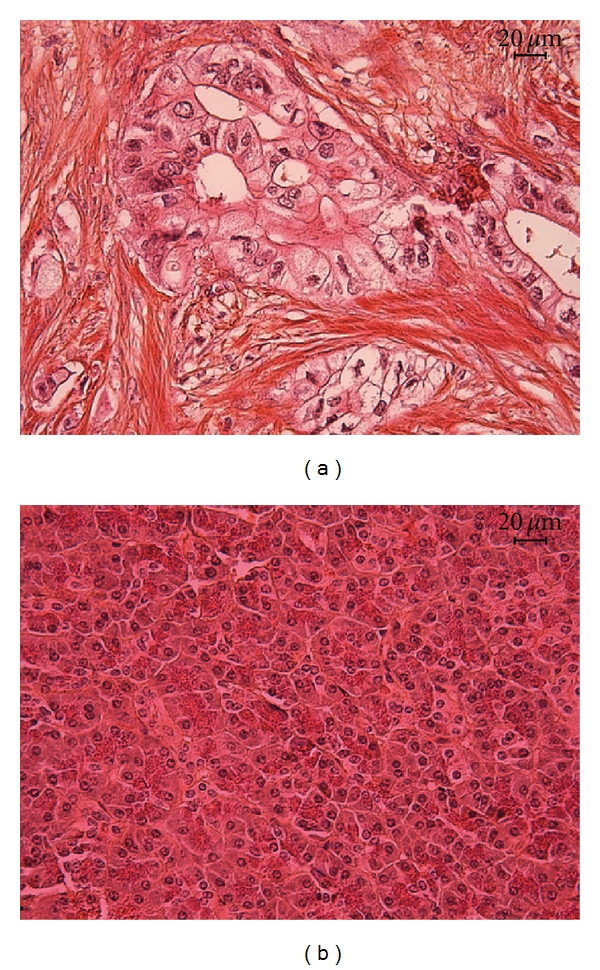
PDAC tissues contain an extensive fibrotic component. Normal pancreas from individuals deceased from hypothermia, and PDAC tissues were stained using Saffron and Hematoxylin. Photographs show the staining for PDAC (a) and normal pancreas (b), the fibrotic tissue is visualized as orange. Size bar 20 *μ*m.

**Figure 3:**
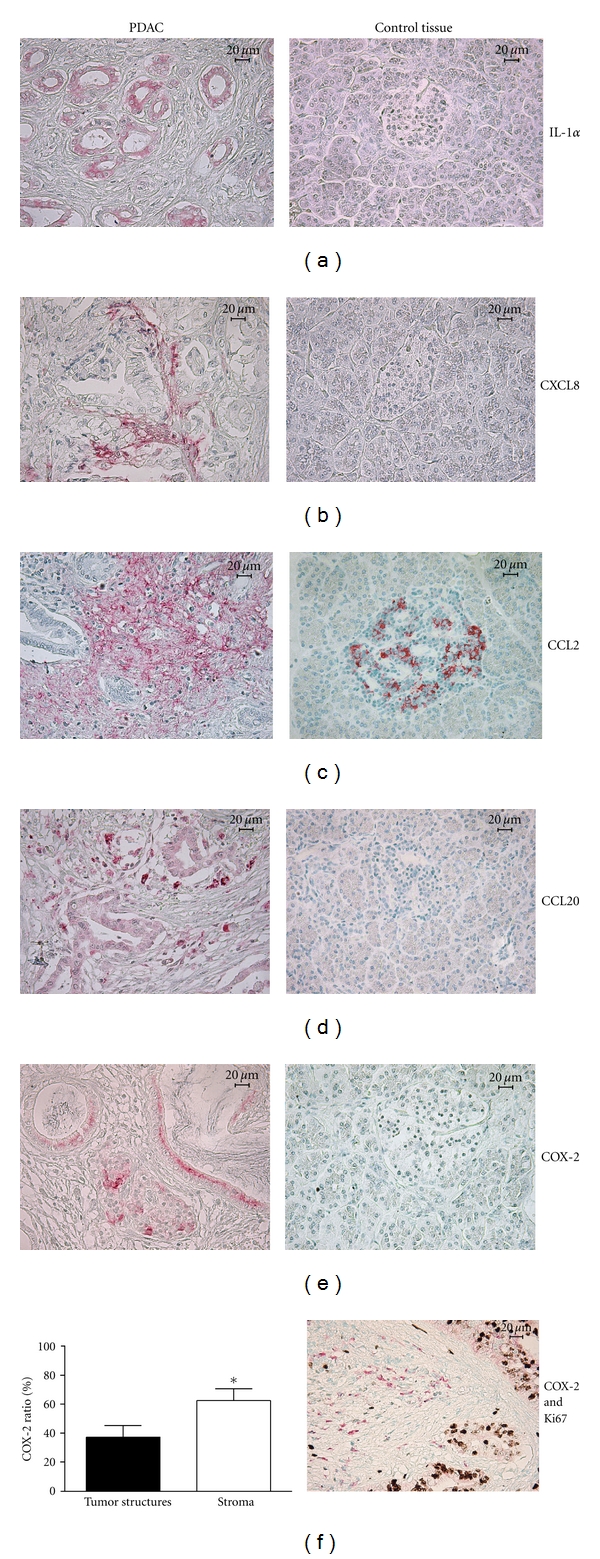
The majority of inflammatory factors in PDAC microenvironment were expressed by the stroma cells. Human PDAC tissue sections were immunostained with anti-COX-2, -CCL20, -CCL2, -CXCL8, and -IL-1*α* antibodies (abs) followed by alkaline phosphatase-conjugated secondary abs and visualized with fast red chromogen. Photographs show the staining (red) for (a) IL-1*α* positive tumor structures, (b) CXCL8 positive tumor stroma, (c) CCL2 positive fibrotic cells, (d) CCL20 positive tumor and stromal cells, and (e) COX-2 positive tumor cells and stroma. Size bar 20 *μ*m. (f) To evaluate the ratio between tumor cell and stroma expressed COX-2, PDAC tissue sections were immunohistochemically stained with anti-COX-2 (red) and anti-Ki-67 abs (brown), followed by alkaline phosphatase- and HRP-conjugated secondary abs and detected using fast red chromogen and DAB, respectively. Areas of double positive cells (tumor nests) were compared to nonproliferative COX-2 positive stroma using a computerized image processing analysis system. *P < 0.05.

The position of the inflammatory factors detected in the tumor microenvironment was done by immunohistochemistry (IHC). We stained PDAC and normal pancreatic tissues with Saffron and Hematoxylin, known to visualize fibrotic tissues. This staining demonstrated that the tumor cells were surrounded by a massive desmoplastic stroma in PDAC (Figures 2(a) and 2(b)). IL-1*α* was exclusively located in tumor cells while normal pancreatic tissue stained negative (Figure 3(a)). CCL2 was expressed by fibrotic stroma and Langerhans islets, in both PDAC and healthy pancreatic tissues. CXCL8 and CCL20 were exclusively expressed by PDAC tissue and mainly localized to the stroma cells, but CCL20 was also detected in tumor cells (Figures 3(b)–3(d)). We found COX-2 expression to be restricted to PDAC samples, where several cell types, for example, tumor cells, CAFs, Langerhans Islet cells, and infiltrating immune cells in the tumor expressed this inflammatory mediator with significantly higher expression in the stroma than in tumor nests (*P* = 0.028) (Figures 3(e) and 3(f)). These findings indicate that the stroma, that is, nonneoplastic tissue, constitutes an important contributor to the inflammation seen in the tumor microenvironment in PDAC.

### 3.3. PDAC Tissue Shows Elevated Gene Expression of Markers Associated with the Expression of Macrophages and Dendritic Cell Subtypes

**Figure 4:**
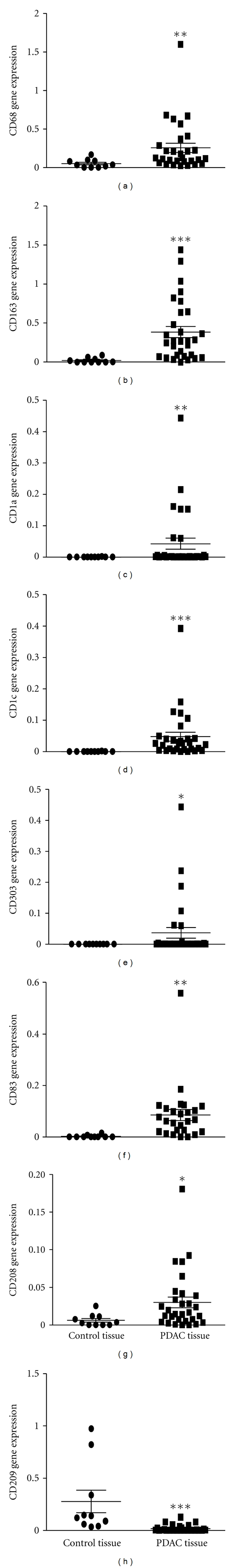
Elevated gene expression levels of markers of infiltrating immune cells in PDAC tissue. RNA was obtained from PDAC (*n* = 30) and normal pancreatic tissue samples (*n* = 10) and assessed for relative gene expression levels of markers expressed by (a and b) macrophages (CD68 and CD163), (c and d) myeloid dendritic cells (CD1a and CD1c), (e) plasmacytoid dendritic cells (CD303), and (f–h) DC maturation/activation status markers (CD83, CD208 and CD209). *P < 0.05, **P < 0.05, ***P < 0.001.

The presence and activation status of tumor infiltrating immune cells was assessed by qPCR using cell-specific markers unique for DCs, or macrophages. We found significantly increased levels of macrophages as measured by CD163 (*P* < 0.001) (Figure 4(a)) and CD68 (*P* < 0.001) (Figure 4(b)), myeloid DCs as measured by CD1a (*P* = 0.032) and CD1c (*P* < 0.001) (Figures 4(c) and 4(d)), and plasmacytoid DCs as measured by CD303 (*P* = 0.007) (Figure 4(e)). The DCs displayed an activated phenotype with significantly increased CD83 (*P* < 0.001), CD208 (*P* = 0.008), and decreased CD209 (*P* < 0.001) (Figures 4(f)–4(h)). Of note, we cannot rule out that a fraction of the CD209 expression detected is due to other cells than DCs as a small subpopulation of tissue macrophages can express this lectin [26].

### 3.4. Enhanced Levels of Infiltrating Immune Cells Such As Macrophages, Dendritic Cells, and Cytotoxic T Cells in the PDAC Stroma

**Figure 5:**
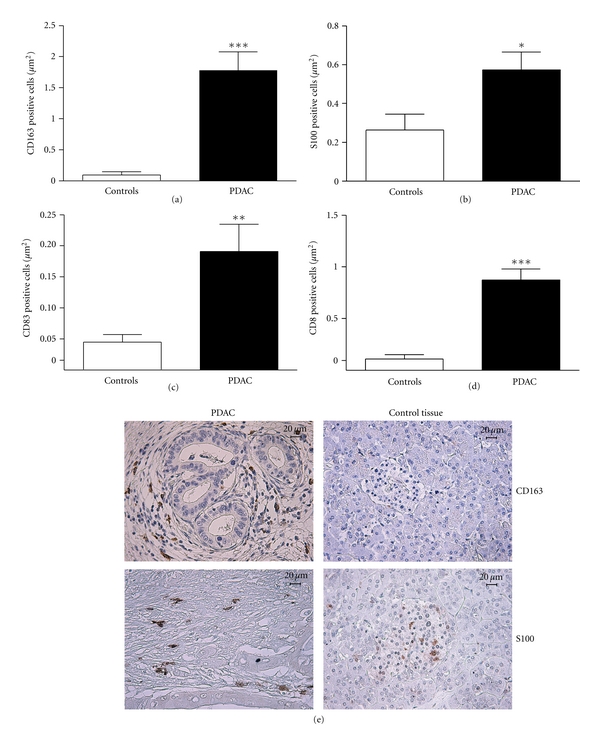
PDAC tissues show infiltration of immune cells such as macrophages, dendritic cells, and CTLs. PDAC and healthy pancreatic tissue samples were immunohistochemically stained with anti CD163, S100, CD83, and CD8 antibodies (abs) followed by biotin-conjugated secondary abs and streptavidin-HRP complex. Peroxidase was detected using DAB chromogen. The amount CD163 (a), S100 (b), CD83 (c), and CD8 (d) positive cells/*μ*m^2^ were calculated from 20 randomly selected fields using a computerized image processing analysis system linked to a microscope. (e) The micrographs show expression and location of CD163, S100, CD83, and CD8 positive cells in the PDAC and healthy pancreatic tissues. Size bar 20 *μ*m. *P < 0.05, **P < 0.05, ***P < 0.001.

We assessed the presence of macrophages, DCs, mature DCs, and cytotoxic T cells in the PDAC microenvironment by immunostaining. Infiltrating CD163 positive macrophages were found in the tumor stroma, and the levels were significantly higher than in the control group (*P* < 0.001) (Figures  5(a) and 5(e)). S100 positive DCs were significantly increased in PDAC compared to normal pancreas (*P* = 0.018). The majority of S100 positive cells in PDAC tissue were located in the fibrous stroma, often in close relation to tumor nests, whereas DCs in healthy pancreas tissues were mainly found in the Langerhans islets (Figures  5(b) and 5(e)). The infiltration of CD83 positive mature DCs varied from low to massive between the different PDAC patients but showed significantly increased numbers compared to healthy pancreas tissue (*P* = 0.004). The highest extent of CD83 positive dendritic cells in PDAC were found in the fibrous stroma (Figures 5(c) and 5(e)). Cytotoxic CD8+ T cells (CTLs) were not found in healthy pancreas, but PDAC tissues had an infiltration of CD8+ T cells in the fibrous stroma surrounding the tumor nests. The numbers of CD8+ T cells found in PDAC samples were significantly higher than in controls (*P* < 0.001) (Figures  5(d) and 5(e)). To make sure that ischemia induced by the surgical procedure was not influencing the amount of infiltrating immune cells in the tissue samples, we compared Whipple resected pancreatic tissue from patients with cystic lesions; tumor adjacent pancreatic tissue (with normal histology) as well as pancreatic tissues obtained from individuals deceased from hypothermia (data not shown). We did not see any difference in the amount of inflammatory cells or expression levels of inflammatory markers for patients with or without stent (data not shown), which is supported by the literature showing only superficial infiltration of inflammatory cells at the location of nondrug delivery stents [27–29]. To eliminate jaundice as a factor modulating the immune cells in our study, we compared the patients' blood bilirubin levels with the levels of the immune cell markers assessed in this study and found no correlation.

### 3.5. PDAC Patients with Higher Levels of Dendritic Cells and Macrophages with CD163 Dominating Phenotype Had the Longest Survival Time

**Figure 6:**
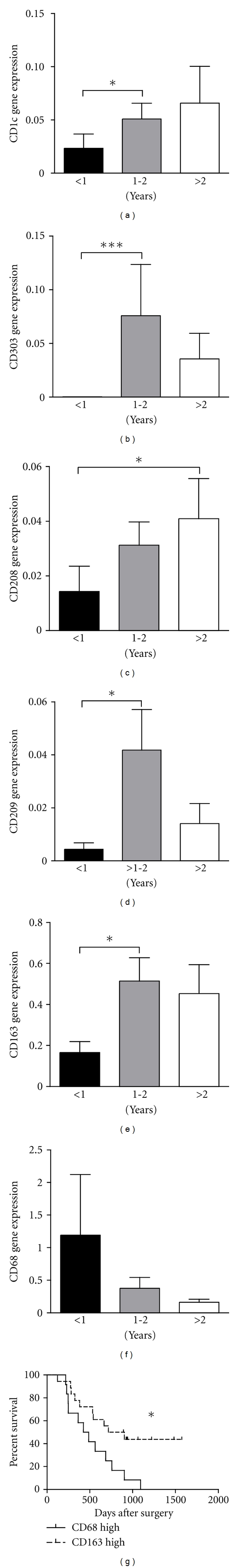
The expression levels of dendritic cell and macrophage markers might predict the PDAC patient survival. The PDAC patients were divided into three groups based on their survival time after tumor resection (less than one year (*n* = 9), between 1 and 2 years (*n* = 10), and more than two years (*n* = 11)). The gene expressions of CD1c (a), CD303 (b), CD208 (c), CD209 (d), CD163 (e), and CD68 (f) were compared between each group. The patients were also divided into two groups based on if the main expression of macrophage markers was CD68 (*n* = 12) or CD163 (*n* = 18) (g). Log-rank (Mantel Cox) test was used for calculation of *P* value. *P < 0.05, **P < 0.05, ***P < 0.001.

The impacts DCs and macrophages have on patient survival were tested by dividing the patients into three groups based on the survival time after tumor resection (short = less than one year (*n* = 9), moderate = between 1 and 2 years (*n* = 10) and long = more than two years (*n* = 11)). The short-time survivors expressed significantly lower gene levels of myeloid DC (CD1c+) and plasmacytoid DC (CD303+) markers as compared to the moderate survivors (*P* = 0.017 (CD1c) and *P* < 0.001 (CD303)). Higher gene expression levels were also observed among the long time survivors, but the difference was not significant (Figures 6(a) and 6(b)). The number of S100 positive DCs in PDAC tissue was higher in the patients surviving more than 2 years compared to patients surviving less than one year, but the difference was not significant (*P* = 0.06) (data not shown). The gene expression levels of the DC activation markers CD208 and CD209 showed higher levels of both tumor infiltrated mature and immature DCs among the moderate (*P* = 0.07 (CD208) and *P* = 0.008 (CD209)) and long-time survivors (*P* = 0.012 (CD208) and *P* = ns (CD209)) as compared to short-time survivors (Figures 6(c) and 6(d)). The short time survivors were also found to have tumors expressing the lowest levels of the macrophage marker CD163 compared to the moderate survivors (*P* = 0.014), and the same tendency was observed among the long-time survivors but the difference was not significant (Figure 6(e)). The tumor expression levels of CD68, another macrophage marker, indicated the long-time survivors to express the lowest levels of CD68+ macrophages (Figure 6(f)). To further investigate the clinical outcome of the expression of CD68 and CD163 by macrophages, we divided the patients into two groups based on if their main gene expression was CD68 or CD163. Patients with a dominating gene expression of CD163 were presented with significantly better clinical outcome than patients with a CD68 dominating macrophage population (*P* = 0.017) (Figure 6(g)).

## 4. Discussion

The composition of the tumor microenvironment is essential for the tumor development and will influence the ability of the immune system to mount a defense against the tumor. PDAC tissues contained several types of inflammatory immune cells, that is, macrophages, MDCs, PDCs, and CTLs, besides high levels of inflammatory factors including IL-1*α*, IL-6, COX-2, TGF*β*, CXCL8, CCL2, and CCL20. The inflammatory factors produced by tumor and stroma cells, including immune cells, CAFs, and Langerhans islet cells, create an environment that could support survival and progression of the malignant cells by altering the inflammatory balance in favor of the tumor. 

In healthy human pancreas the Langerhans islets were found to be the main reservoir of DCs and, to our knowledge, this has previously only been reported in mice [30]. An obvious relocation of DCs was seen in the PDAC tissues, where most DCs were located in the fibrotic stroma. The expression of DCs were found to be higher in the tumors than in normal healthy pancreas, but in general the PDAC tumors showed very low levels of both MDCs and PDCs, and a shortage of DCs, mature or immature, was associated with poor clinical outcome. The low levels of DCs and the location in the tumor stroma confirmed previous findings by Dallal et al. [31], but with the use of gene-specific markers we have further extended these findings to include immunomodulatory and chemotactic factors. Furthermore, we were able to show a connection between the DC infiltration and the clinical outcome of the patients. 

The chemokines produced by the fibrotic stroma in PDAC tumors, including CXCL8 and CCL20, have previously been shown to initiate the migration of DCs to this site [32]. Moreover, CXCL8 derived from tumor cells have been shown to retain DCs in the tumor resulting in deficient migration to the lymph nodes and also impaired immune response against the cancer [33]. This is also supported by our findings of elevated levels of mature DCs expressing the phenotypic maturation markers CD83 and CD208 in addition to decreased levels of CD209. Nevertheless, patients with low levels of infiltrating CD208 positive DCs had the shortest survival time among the PDAC patients which is in accordance with findings in melanoma [34]. This could indicate an important role for the composition of the inflammatory tumor microenvironment and its ability to retain and mature the DCs.

PDCs are normally found in blood but can also be found at sites of chronic inflammation including cancers [35]. PDAC tumors have been shown to express high levels of CXCL12 and CCL2, which could promote PDC migration into the tumor [36, 37]. This is in accordance with our data showing the presence of PDCs in PDAC tissue and the lack of PDCs in healthy pancreatic tissue. 

IDO expressed by DCs or cancer cells have been shown to suppress the immune response to tumors by establishing immunological tolerance [38]. Furthermore, the expression of IDO in ovarian, endometrial, and colon cancer has been correlated to poor clinical outcome [39]. The COX-2 product PGE_2_ is known to be an inducer of IDO expression in antigen presenting cells and inhibition of COX-2 expression both in vitro and in vivo reduced the expression of IDO [40]. This is in accordance with our data showing increased expression of both COX-2 and IDO in the PDAC tissues. Consequently, the elevated levels of IDO detected in the PDAC tumors could be a contributing factor to the lack of an efficient immune response against the tumor.

Another factor associated with immune tolerance, TGF*β*, expressed by tumor cells and tumor infiltrating DCs has been shown to promote the expansion of natural occurring Tregs (nTregs) [41, 42]. In the present study, PDAC but not healthy pancreatic tissue expressed TGF*β* which might contribute to the immune suppression by promoting the expansion of nTregs in the tumor microenvironment and this needs further investigation. 

The negative immunoregulatory receptor PD1, expressed by activated T cells, was detected in 40% of the PDAC tumors. The presence of PD1 positive immune cells has been shown to be associated with adverse pathology and poor outcome in patients with renal cell carcinoma [43, 44] and might also be involved in the impaired immune response against PDAC tumors. 

Macrophages are derived from progenitors, that is, monocytes existing in the circulation, and are recruited to tissues under the influence of CCL2 [45], which was found elevated in PDAC tissue in the present study. Macrophages may enhance the tumor growth as they secrete VEGF-A, VEGF-C, and FGF, which are known to contribute to angiogenesis in tumor and also to increase the metastatic potential of tumor cells [9]. When infiltrating the tumor microenvironment, these cells are referred to as tumor-associated macrophages (TAMs). TAMs are important inflammatory cells correlating with tumor progression and bad prognosis in, for example, breast, lung, and cervix cancer {Leek, 2000 #160; Zhang, #157; Pollard, 2004 #151}. Our data suggest that high gene expression levels of CD68 might be associated with poor prognosis, though nonsignificant, while high levels of CD163 were found among the patients with the best clinical outcome, which could point toward the presence of two different types of TAMs with opposite functions. This was supported by our findings pointing to a survival advantage for patients with a CD163 dominating macrophage phenotype. Moreover, the PDAC tumors expressed macrophage markers to a higher extent than DC markers which might at least partly be explained by the increased expression of IL-6 which has been shown by Chomarat et al. [46] to promote the differentiation of monocytes into macrophages at the expense of DCs [46]. We have previously identified IL-1*α* to be the main tool used by the tumor cells to activate CAFs to produce several inflammatory factors (e.g., IL-6, CCL20, CXCL8, COX-2, and VEGF-A), and this could be one mechanism the tumor cells use to escape elimination by the immune system. Blocking the IL-1 signaling cascade using synthetic IL-1RA (Kineret) drastically reduced the expression of the inflammatory factors in vitro [47], and treatment with IL-1 antagonist might thus have the potential to downregulate the levels of immunomodulatory factors in PDAC tumors. 

This study points to the importance of the fibrotic stroma in the production of inflammatory factors and accommodation of immune cells in PDAC tumors. Therapies targeting the desmoplastic stroma and/or inflammatory factors such as IL-6, COX-2, CXCL8, and TGF*β* might have the potential to manipulate the tumor microenvironment to benefit attraction of DCs and differentiation of monocytes into functional DCs which could affect the clinical outcome for the PDAC patients.
